# Large but variable methane production in anoxic freshwater sediment upon addition of allochthonous and autochthonous organic matter

**DOI:** 10.1002/lno.10786

**Published:** 2018-02-06

**Authors:** Charlotte Grasset, Raquel Mendonça, Gabriella Villamor Saucedo, David Bastviken, Fabio Roland, Sebastian Sobek

**Affiliations:** ^1^ Laboratory of Aquatic Ecology, Department of Biology Federal University of Juiz de Fora Juiz de Fora Brazil; ^2^ Limnology, Department of Ecology and Genetics Uppsala University Uppsala Sweden; ^3^ Department of Thematic Studies – Environmental Change Linköping University Linköping Sweden

## Abstract

An important question in the context of climate change is to understand how CH_4_ production is regulated in anoxic sediments of lakes and reservoirs. The type of organic carbon (OC) present in lakes is a key factor controlling CH_4_ production at anoxic conditions, but the studies investigating the methanogenic potential of the main OC types are fragmented. We incubated different types of allochthonous OC (alloOC; terrestrial plant leaves) and autochthonous OC (autoOC; phytoplankton and two aquatic plants species) in an anoxic sediment during 130 d. We tested if (1) the supply of fresh alloOC and autoOC to an anoxic refractory sediment would fuel CH_4_ production and if (2) autoOC would decompose faster than alloOC. The addition of fresh OC greatly increased CH_4_ production and the δ^13^C‐CH_4_ partitioning indicated that CH_4_ originated exclusively from the fresh OC. The large CH_4_ production in an anoxic sediment fueled by alloOC is a new finding which indicates that all systems with anoxic conditions and high sedimentation rates have the potential to be CH_4_ emitters. The autoOC decomposed faster than alloOC, but the total CH_4_ production was not higher for all autoOC types, one aquatic plant species having values as low as the terrestrial leaves, and the other one having values as high as phytoplankton. Our study is the first to report such variability, suggesting that the extent to which C fixed by aquatic plants is emitted as greenhouse gases or buried as OC in sediment could more generally differ between aquatic vegetation types.

Lakes and reservoirs are important sources of the greenhouse gases (GHG), carbon dioxide (CO_2_) and methane (CH_4_), to the atmosphere (Cole et al. 2007; Tranvik et al. 2009; Bastviken et al. 2011). CH_4_, which is produced during the anoxic decomposition of organic carbon (OC), is of particular interest since it has a warming potential 28 times higher than CO_2_ (IPCC 2014). CH_4_ production is mainly occurring in the sediments, where oxygen is usually limited to the upper millimeters (Sobek et al. 2009, 2012). Even though a significant part of the produced CH_4_ may be oxidized into CO_2_ and not be emitted to the atmosphere, there seems to be a strong correlation between CH_4_ production and emission (Yvon‐Durocher et al. 2014). In addition, CH_4_ production is strongly dependent on temperature (Bastviken 2009; Yvon‐Durocher et al. 2014), such that tropical freshwaters may be particularly strong CH_4_ sources (Tranvik et al. 2009; Bastviken et al. 2010). Tropical hydropower reservoirs have been pointed as strong anthropogenic CH_4_ sources (Barros et al. 2011), which is of imminent concern given the current boom in hydropower construction in many tropical countries (Zarfl et al. 2015). Therefore, an important question in the context of climate change as well as sustainable energy production is to understand how CH_4_ production is regulated in anoxic sediments of lakes and reservoirs.

OC decomposition and associated CH_4_ production under anoxic conditions is strongly controlled by the types of OC present (Sobek et al. 2009; Gudasz et al. 2012). Labile compounds are expected to be readily decomposed under anoxic conditions, whereas the decomposition of more complex compounds might be limited by low hydrolysis and fermentation rates (Zehnder and Svensson 1986; Valentine et al. 1994; Kristensen et al. 1995; Bastviken et al. 2003). In lakes, allochthonous OC (alloOC, i.e., OC derived from land) is usually assumed to have a lower reactivity than autochthonous OC (autoOC, i.e., OC derived from aquatic production) because compared to aquatic plants and phytoplankton, terrestrial plants have more support tissues, rich in complex structural compounds (Rascio 2002; Dai et al. 2005). Phytoplankton and other algae are supposed to be the most labile autoOC sources because they contain almost no support tissues (Kankaala et al. 2003; Dai et al. 2005). There is evidence that at anoxic conditions, the decomposition of alloOC could be limited in comparison to autoOC (Kristensen and Holmer 2001; Sobek et al. 2009; West et al. 2012). Congruently, recent studies demonstrated a correlation between autoOC (and thereby the lake‐internal primary production) and CH_4_ production and emissions (Deemer et al. 2016; DelSontro et al. 2016; West et al. 2016). However, while some eutrophic lakes are dominated by phytoplankton, others are dominated by macrophytes, but the effect of these different types of autoOC on lake CH_4_ production and emission is currently unknown. In addition, many lakes have little autoOC production but receive large amounts of alloOC from their catchments, which also might affect CH_4_ production and emission (West et al. 2012; Brett et al. 2017). Current knowledge on the methanogenic potential of different types of OC in lake sediments are fragmented because they typically include only one (Schulz and Conrad 1995; Schwarz et al. 2008) or, more rarely, two (Kankaala et al. 2003; West et al. 2012) of the three main types of OC occurring in lakes, i.e., phytoplankton, aquatic vascular plants, and terrestrial vascular plants, respectively. Hence, there is at present no comprehensive understanding of the effects of productivity, dominating aquatic vegetation type, and terrestrial OC input on CH_4_ emissions from lakes.

While the studies cited above deal with the effect on CH_4_ production of newly added OC to sediment, the sediment which receives these inputs of new OC already constitutes a large OC pool and a potential CH_4_ source. Studies have reported very low sediment CH_4_ production rates from lakes across different latitudes (Schwarz et al. 2008; Conrad et al. 2011; West et al. 2012), if compared to what is obtained after fresh OC addition (Schwarz et al. 2008; West et al. 2012), pointing toward a low importance of the residing sediment OC pool for CH_4_ production. However, the contribution of the residing lake sediment OC pool to CH_4_ following fresh OC addition has never been assessed. At oxic conditions, several studies suggest that the decomposition of refractory sediment OC tends to be stimulated by the addition of labile OC, through an effect called “positive priming” (Guenet et al. 2010, 2014). Studies in anoxic soils on the relative contribution of soil OC and fresh OC to CH_4_ production returned contrasting results: the application of fresh OC could either enhance (Chidthaisong and Watanabe 1997; Lu et al. 2000; Ye et al. 2016) or decrease (Conrad et al. 2012) CH_4_ production derived from soil OC. Therefore, it is at present not possible to gauge the contribution of the residing lake sediment OC pool to CH_4_ production following fresh OC addition, calling for studies that partition the sources of CH_4_ during the anoxic decomposition of fresh OC in lake sediments.

In this study, we hypothesized that (1) the supply of fresh OC to an anoxic refractory sediment will increase CH_4_ production and that CH_4_ production will mainly be fueled by fresh OC, (2) autoOC will decompose faster than alloOC and thus will sustain higher CO_2_ and CH_4_ production rates. For that, we incubated several types of allochthonous and autochthonous organic matter together with a refractory sediment under anoxic conditions, and monitored the production and isotopic composition of CO_2_ and CH_4_ over a 130 d period.

## Materials and methods

### Overview

We performed anoxic incubations of sediment from a drinking water reservoir with and without additions of OC from various sources. Four different types of OC were added to the sediment: aquatic plant leaves from two different species, phytoplankton, and a mixture of land plant leaves. The following part describes first the collection and the analyses (total carbon (TC), total nitrogen (TN), δ^13^C) of the materials used for the incubation experiment. We then describe the monitoring of CO_2_, CH_4_, and O_2_ in the headspace during the incubation experiment, and the calculations of the cumulative TCO_2_ (headspace CO_2_ + dissolved inorganic carbon (DIC)) and CH_4_ concentrations, as well as of the OC remaining after C mass loss during degradation. An exponential decay model was applied to the remaining OC to compare the dynamics of decomposition between the different added types of OC. Finally, we describe how CO_2_ and CH_4_ were analyzed for δ^13^C and the method and calculations used to partition the OC sources fueling CH_4_ production during incubation.

### Experimental scheme

The different potential sources for methanogenesis (sediment and different types of added OC) were sampled as follows.

#### Sediment

The sediment was sampled in an oligotrophic drinking water reservoir situated in the sub‐tropical Atlantic Forest region of Brazil (Chapéu d'Uvas, 21°35′1.54″S, 43°31′42.37″W; mean total phosphorus (TP) 12 μg L^−1^ and mean TN 452 μg L^−1^; J. Paranaíba et al. 2018). The sediment was collected near the entrance of the river, where the allochthonous sediment deposition is high (A. Isidorova et al. unpubl.). Three (3) cores were sampled with a gravity corer and the 3–4 uppermost cm of sediment, considered the most active for organic matter decomposition, were sampled by slicing, mixed, and used for the experiment.

#### OC additions

Senescent leaves of 17 different tree and shrub species, having contrasting thickness and size, were collected close to the reservoir, in order to be used as an alloOC source in the experiment. Leaves were cut to approximately 1 cm^2^ and mixed. As autoOC sources, we used two different aquatic plants and phytoplankton. Senescent leaves of two C3 aquatic plant species, *Salvinia auriculata*, a free floating species, and *Nymphoides indica*, a rooted species with floating leaves, both common in Central and South‐American lakes (Mortillaro et al. 2011; Mendonça et al. 2013), were collected in two other reservoirs of the Atlantic Forest region of Brazil (Simplício, 22°05′38.7″S, 43°04′17.7″W; João Penido, 21°39′48.5″S, 43°23′18.7″W for *S. auriculata* and *N. indica*, respectively). The leaves were washed with tap water to remove sediment and invertebrates and for *N. indica*, the leaves were also cut to ca. 1 cm^2^, which is approximately the size of *S. auriculata*'s leaves. Phytoplankton was collected during a bloom in another reservoir in the Atlantic Forest region (Funil, 22°31′45.47″S, 44°34′2.25″W) with a 20 μm plankton net. The species were identified as a mixture of the blue green algae *Microcystis aeruginosa, Dolichospermum* sp., and *Cylindrospermopsis raciborskii*.

#### Artificial lake water and sediment inoculum

Artificial lake water enriched in TN and TP (15.8 μg L^−1^ of KH_2_PO_4_ and 4.57 mg L^−1^ of NH_4_NO_3_) was prepared according to Attermeyer et al. (2014) and used for all treatments. Since each added OC source and the sediment were sampled at different sites, different microbes might have been present, and also, the microbial community present in the sediment of the oligotrophic reservoir might not have been efficient to decompose the different OC types (Leflaive et al. 2008; Comte and Del Giorgio 2009). To avoid these possible effects, one sediment core was sampled in each of the three reservoirs used for aquatic plant and phytoplankton collection and the upper layers (3–4 cm) of each core were mixed in equivalent proportions to constitute a sediment inoculum.

All materials (sediment and added OC) were incubated fresh as drying affects the decomposition dynamics (Gessner 1991), and were stored for 5 d maximum in the dark at 4°C before incubation. The phytoplankton was also considered senescent as it usually takes 5–10 d for cyanobacteria to die in the dark (Furusato and Asaeda 2009). The incubation experiment with autoOC began in March 2015 and the incubations with alloOC began in April 2015 thus the sediment sampling occurred at two dates, in March and in April 2015.

**Figure 1:**
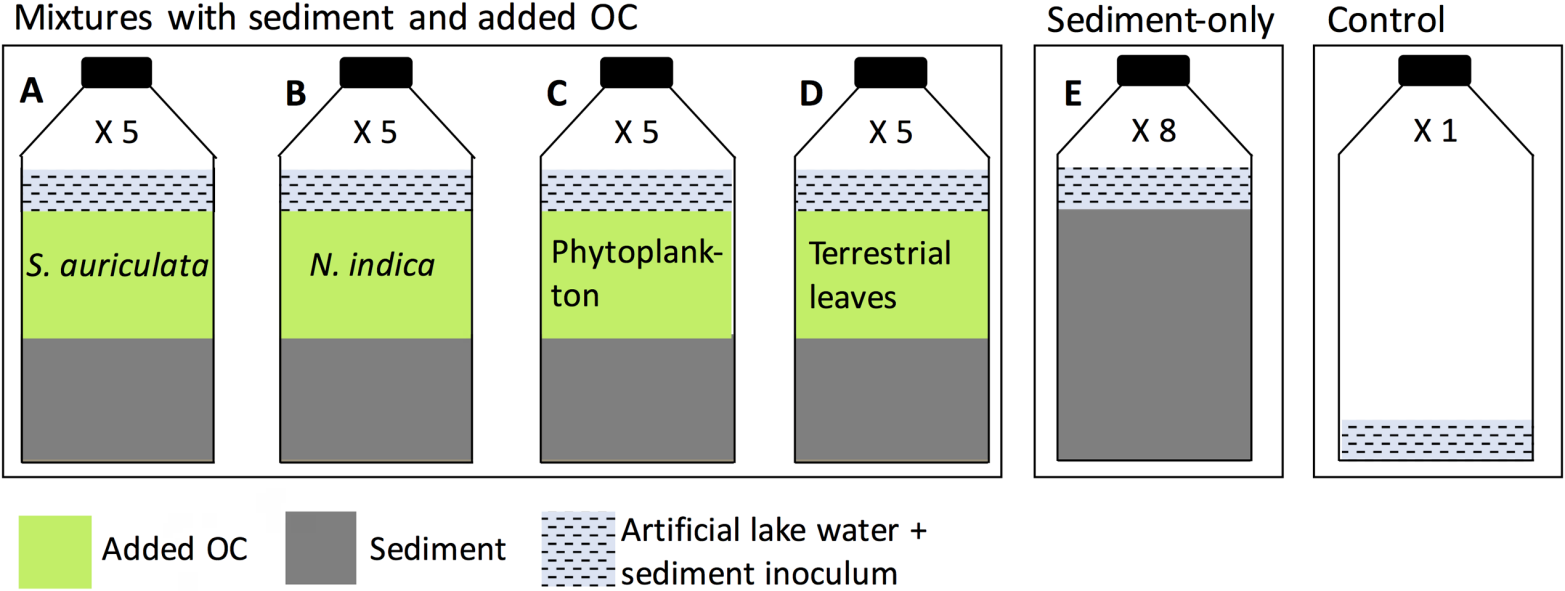
Experimental scheme. See text for details. Among the five replicates of the mixture treatments (A–D), three replicates were primarily used for isotopic measurements, and two replicates were used exclusively for CO_2_ and CH_4_ concentration measurements. For the sediment‐only treatment (E), five replicates were filled with the sediment sampled in March and three with the sediment sampled in April.

We incubated five different treatments: (A) sediment mixed with *S. auriculata*, (B) sediment mixed with *N. indica*, (C) sediment mixed with phytoplankton, (D) sediment mixed with terrestrial leaves, and (E) sediment without any OC addition (Fig. 1). One hundred milliliter glass serum bottles were filled with treatment material, 30 mL of artificial lake water and two drops of the sediment inoculum. The mixture treatments (A–D) contained 18.6 mg C (phytoplankton) to 40.6 mg C (*S. auriculata*) of added OC source, plus 24.4 ± 4.2 mg C of sediment, and the treatment with sediment‐only (E) contained 47.8 ± 8.3 mg C (Table 1). All treatments were incubated in five replicates except for treatment E (sediment‐only) which was incubated in five replicates with the sediment sampled in March, and three replicates with the sediment sampled in April. One control was prepared with the artificial lake water and sediment inoculum only (Fig. 1).

**Table 1:** Characteristics of the added OC and sediment.

	TC (%)^a^	TN (%)^a^	C/N	δ^13^C‐OC (‰)^a^	Quantity (mg C) in the mixtures^b^	Quantity of added OC/quantity of sediment OC
*S. auriculata*	34.8	1.7	20.5	−28.9	40.6 ± 1.3	1.9
*N. indica*	41.0	1.0	40.0	−27.8	27.3 ± 0.9	1.3
Phytoplankton	44.8	8.4	5.4	−16.8	18.6 ± 0.6	0.9
Terrestrial leaves	45.2	1.1	41.1	−30.5	32.4 ± 4.8	1.1
Sediment	2.2	0.2	10.6	−22.8	24.4 ± 4.2	—

For sediment, δ^13^C‐OC was equivalent to δ^13^C of TC because solid carbonate content was negligible.

a *n* = 4 for sediment (the sediment sampled in April and in March are pooled in this table because of their similar characteristics), *n* = 2 for phytoplankton and terrestrial leaves, and *n* = 1 for *N. indica* and *S. auriculata*. The maximum standard errors were 0.6% for TC, 0.06% for TN, and 0.6 for δ^13^C‐OC.

b Mean ± SD.

To create anaerobic conditions, the bottles were initially flushed with N_2_ and then closed with gas‐tight butyl‐rubber septa (thickness of 12 mm) and aluminum crimp seals. The bottles were flushed again 24 h after closing to remove any O_2_ trace (Conrad et al. 2010), and this day was considered day 0 of the experiment. The bottles were then kept in the dark to avoid photosynthesis, at a temperature between 20°C and 22°C and without agitating, as that may affect syntrophic microbial associations and thus methanogenesis (Dannenberg et al. 1997; Guerin et al. 2008). During the incubation, the headspace gas was sampled for CH_4_ and CO_2_ concentration or δ^13^C measurement at several dates with a plastic syringe equipped with a three‐way valve. As oversampling may reduce the headspace gas pressure inside the bottles and lead to contamination of headspace with air, we sampled relatively small volumes of headspace (between 0.5 mL and 2 mL) and divided the replicates for gas concentration or for isotopic analyses to limit the number of samplings per bottle. The bottles were flushed twice with N_2_, at days 30 and 121 (first batch) or 103 (second batch), to restore atmospheric pressure and to avoid methanogenesis inhibition which can be caused by the accumulation CH_4_, CO_2_, or other volatile metabolic end products in the headspace (Magnusson 1993; Guerin et al. 2008). We sampled approximately the same amount of gas inside all the replicate bottles, ca. 7 mL, before the first flushing with N_2_, and ca. 6 mL between the first and the second flushing. Two of the five replicates per treatment were used exclusively for the gas concentration measurement until day 60, when they were opened for pH measurement. The three other replicates were used primarily for δ^13^C‐CH_4_ and δ^13^C‐CO_2_. The gas concentrations in the three replicates used for δ^13^C‐CH_4_ and δ^13^C‐CO_2_, were measured at days 30 and 60, at the same time than for the other replicates, and after day 100. The first batch with autoOC (treatments A–C) was incubated for 136 d and the second batch with alloOC (treatment D) for 118 d.

### Analyses of added OC and sediment (TC, TN, and δ^13^C)

A part of the materials prepared for the incubation was used for elemental and isotope analyses, dried in the oven at 70°C during 48–72 h and ground with a mortar and a pestle, or finely cut with scissors and then ground, when grinding was difficult (*S. auriculata* and terrestrial leaves). The dry material of each OC type was weighed (ca. 5 mg of plant leaves or phytoplankton, and 50 mg of sediment) into separate tin capsules for TC, TN, and δ^13^C analyses. In addition, for sediment, OC content and its δ^13^C signature were measured after removing inorganic carbon by the addition of acid (20 μL of deionized water and 150 μL of HCl 5%) to ca. 50 mg of sediment samples in silver capsules and after overnight drying at 50°C (Brodie et al. 2011; Karlsson et al. 2011). Carbonate content in sediment was calculated from the difference between TC and OC contents. Plant and phytoplankton samples were not acidified as they are low in carbonates and because acidification may affect OC content and its δ^13^C (Brodie et al. 2011; Burke et al. 2015). TC, TN, and δ^13^C were measured with an elemental analyzer coupled to a mass spectrometer (Europa Hydra 20/20, University of California, Davis, Stable Isotope Facility, Davis, California, U.S.A.).

### O_2_, TCO_2_, CH_4_, and remaining OC

Gaseous O_2_ concentrations were monitored during the incubation with an optical sensor system and noninvasive oxygen sensor spots (Fibox 4 and PSt3, PreSens–Precision Sensing GmbH, Regensburg, Germany). For all treatments, anoxic conditions were reached and maintained throughout the experiment.

CO_2_ and CH_4_ concentrations in the headspace of the bottles were measured by intracavity laser absorption spectroscopy with an Ultra‐Portable Gas Analyzer (Los Gatos Research, Mountain View, California, U.S.A.) using a discrete sample measurement method adapted from Gonzalez‐Valencia et al. (2014). The gas analyzer was equipped with a gas‐tight custom‐made sample inlet and a water filter (pore size 1 *μ*m, Millipore, Eschborn, Germany). Ambient outdoor air was used as carrier gas, with a CO_2_ absorber containing soda lime connected upstream of the inlet, which decreased the CO_2_ and CH_4_ baselines to below 1 ppm and 1.8 ppm, respectively. Injections into the sample inlet via a plastic syringe equipped with a three‐way valve led to peaks (concentration in ppm over time) that were integrated with the R software. A calibration curve was made by injecting 0.5–1 mL of gases with known CO_2_ and CH_4_ concentrations, prepared from the dilution of a standard (5.05% of CH_4_ and 20% of CO_2_). For the measurement of CO_2_ and CH_4_ concentrations in the headspace, the bottles were shaken before gas sampling to release CH_4_ bubbles and to equilibrate with the headspace. 0.5–2 mL of gas was sampled in the headspace with the syringe and directly injected into the sample inlet connected to the gas analyzer.

CO_2_ and CH_4_ concentrations in the headspace were converted into molar units according to the ideal gas law. CO_2_ and CH_4_ concentrations in the water were calculated from their concentration in the headspace, the volume of artificial lake water and the water content of the sediment, and the specific gas solubility of CO_2_ (Weiss 1974) and CH_4_ (Yamamoto et al. 1976), respectively. pH was measured at day 0, day 60, and at the end of the incubation (day 118 or day 136) with a benchtop pH meter (Micronal, B474). pH values were stable for all treatments (ca. 6.9) except for the treatments with phytoplankton where pH increased from 6.9 (day 0) to 7.4 (at day 60 and day 136). DIC was calculated from pH, CO_2_ concentrations in the water, and equilibrium constants (Stumm and Morgan 2012). For all treatments except that with phytoplankton, DIC was calculated assuming a constant pH of 6.9. For the treatments with phytoplankton, DIC was calculated making a linear interpolation of pH from 6.9 at day 0 to 7.4 at day 60, and then with a constant pH of 7.4 from day 60 to day 136. The change of total CO_2_ (i.e., both in the headspace and in the water phase as DIC) is noted TCO_2_ production hereafter. Flushing with N_2_ removed on average 94% of CO_2_ and 99% of CH_4_ concentrations. Cumulative TCO_2_ and CH_4_ productions were calculated by adding the concentrations removed by flushing to the concentration measured after flushing and are used throughout the manuscript. TCO_2_ and CH_4_ productions rates were calculated as the difference in TCO_2_ and CH_4_ concentrations between two consecutive dates of concentration measurement (when no flushing occurred between the two dates) divided by the time interval between the two dates.

The remaining amount of OC at time *t* (*C_t_*) was calculated as the subtraction of the initial C mass (*C*_i_) by the C lost as TCO_2_ and CH_4_. Hence, remaining OC included particular OC (POC) as well as dissolved OC (DOC). The production of CO_2_ and CH_4_ over time from added OC were estimated by removing sediment production of CO_2_ and CH_4_ obtained with the sediment‐only treatment (sediment production of CO_2_ and CH_4_ were normalized by the amount of sediment OC present). Remaining OC was divided by the initial C mass to obtain a fraction of remaining OC (*C_t_*/*C*_i_). The initial C mass was that of the added OC for treatments with sediment and added OC, and the initial C mass was that of the sediment for the treatments with sediment‐only. In the same way, TCO_2_ and CH_4_ production over time and production rates were normalized by the initial C mass of added OC for the treatments with sediment and added OC, or by the initial C mass of sediment for the treatments with sediment‐only.

### Exponential decay model of remaining OC

Exponential models are the most common models used for sediment and litter decomposition (Westrich and Berner 1984; Adair et al. 2010; Forney and Rothman 2012). An exponential decay model with a residual pool was therefore fitted to the fraction of remaining OC, in order to compare the dynamics of decomposition between the different mixture treatments (A–D) according to Westrich and Berner (1984):

$$\frac{\;C_{t}}{C_{i}}={a(e^{-k.t})}^{\;}+(1-a)$$

where $\frac{\;C_{t}}{C_{i}}$ is the fraction of remaining OC at time t (unitless), a is the initial fraction of the degradable pool, (1−a) is that of the residual pool (unitless), and k is the first‐order decay constant (i.e., the speed of decay of the degradable pool in d−1). Therefore, a refers to the proportion of the degradable pool while k refers to the reactivity of the degradable pool.

The fraction of remaining OC was fitted to a nonlinear model using generalized least squares (gnls function in package “nlme,” R Core Team 2015). The significance of the parameters estimated from the model ( a and k) was tested with an analysis of variance (ANOVA), and the relevance of the model was checked with visual examination of data against fitted values and with residual plots. We tested if the parameters differed between treatments by comparing different sets of parameter models with the ANOVA method (Ritz and Streibig 2008). The replicates used primarily for δ^13^C‐CH_4_ and δ^13^C‐CO_2_ were not included at days 30 and 60 in the model to limit heteroscedasticity.

### δ^13^C of CO_2_ and CH_4_

δ^13^C of CO_2_ and CH_4_ in the headspace were measured in three replicates of each treatment at days 10, 18, and 40. Measured CO_2_ and CH_4_ concentrations were used to calculate the suitable volume of headspace to sample for isotope analysis. In order to reach the concentration range suitable for analysis, 0.5–2 mL of the headspace was diluted into 5.9–12 mL vials (Soda Glass Vials 819W, Labco, High Wycombe, UK) being pre‐evacuated and thereafter flushed‐filled with N_2_ at atmospheric pressure (Sturm et al. 2015). Analyses were made using a Thermo Scientific GasBench‐Precon interfaced to a Delta V Plus isotope ratio mass spectrometer (ThermoScientific, University of California, Davis, Stable Isotope Facility, Davis, California, U.S.A.).

### CH_4_ source partitioning

The δ^13^C signature of CH_4_ was used to assess how much of the produced CH_4_ was derived from added OC and how much was derived from sediment OC. δ^13^C of CH_4_ mainly depends on the different C fractionation during acetoclastic vs. hydrogenotrophic CH_4_ production and on the δ^13^C signature of the substrates (acetate or CO_2_ + H_2_) used for methanogenesis. The great variability in C fractionation factors associated with methanogenesis (between 10‰ and 70‰) is often a main difficulty partition the sources of CH_4_ (Conrad et al. 2012). Therefore, we used a method which does not rely on the quantification of the C isotopic fractionation factors. The δ^13^C of CH_4_ can be compared between different mixture treatments (treatments with sediment + different added OC) according to Conrad et al. (2012).

For each mixture:

(1) $$\delta {}^{13}{CH}_{4,\;mixture}\;=f_{added\;OC\;}\times \;\delta {}^{13}{CH}_{4,\;added\;OC}\;+\;{(1-f}_{added\;OC\;})\;\times \;\delta {}^{13}{CH}_{4,\;SOC}\;$$

where δCH4, mixture 13 is the measured δ^13^C of CH_4_ from the decomposition of the mixture treatment (added OC + sediment), $f_{added\;OC\;}$ is the contribution of the added OC to the CH_4_ produced, δCH4, added OC 13 is the theoretical δ^13^C of CH_4_ derived from the added OC, and δCH4, SOC 13 the theoretical δ^13^C of CH_4_ derived from the sediment OC.

Since δCH4, added OC 13 is unknown, the formula can be rewritten using ${ɛ}_{{added\;OC,\;CH}_{4}}$, the isotopic enrichment factor involved in the conversion of added OC into CH_4_ (i.e., δCH4, added OC 13=δCadded OC+ ɛadded OC, CH4 13):

(2) $$\delta {}^{13}{CH}_{4,\;mixture}\;=f_{added\;OC\;}\times \;\left(\delta {}^{13}C_{added\;OC}\;+{ɛ}_{{added\;OC,\;CH}_{4}}\right)+{(1-f}_{added\;OC\;})\;\times \;\delta {}^{13}{CH}_{4,\;SOC}$$

We can compare the δCH4, mixture 13 of two different types of OC to determine their contribution to the CH_4_ produced relative to the sediment (1) if we assume the same contribution $f_{added\;OC\;}$ and the same isotopic fractionation factor $\varepsilon_{{added\;OC,\;CH}_{4}}$ for the two types added OC, and (2) if the two types of OC have sufficiently different δCadded OC 13 values. The added OC contribution to the CH_4_ produced may be calculated by subtracting Eq. (2) for the two different types of added OC (Conrad et al. 2012; Ye et al. 2016):

$$\delta {}^{13}{CH}_{4,\;mixture\;1}\;-\;\delta {}^{13}{CH}_{4,\;mixture\;2}\;=$$

$$f_{added\;OC\;1\;}\times \;\left(\delta {}^{13}C_{added\;OC\;1}+\varepsilon_{{added\;OC\;1,\;CH}_{4}}\right)+{(1-f}_{added\;OC\;1\;})\;\times \;\delta {}^{13}{CH}_{4,\;SOC}\;$$

(3) $$-\;f_{added\;OC\;2\;}\times \;\left(\delta {}^{13}C_{added\;OC\;2}\;+\varepsilon_{{added\;OC\;2,\;CH}_{4}}\right)-{(1-f}_{added\;OC\;2\;})\;\times \;\delta {}^{13}{CH}_{4,\;SOC}\;$$

Here, $f_{added\;OC\;1\;}$ = $f_{added\;OC\;2\;}\;=\;f_{added\;OC\;}$ and $\varepsilon_{{added\;OC\;1,\;CH}_{4}}\;=\varepsilon_{{added\;OC\;2,\;CH}_{4}}$.

Hence the contribution (in %) of the added OC to the CH_4_ produced in the mixture treatments is:

(4) $${f_{added\;OC\;}=}_{\;}\frac{\delta {}^{13}{CH}_{4,\;mixture\;1}\;-\;\delta {}^{13}{CH}_{4,\;mixture\;2}\;}{\delta {}^{13}C_{added\;OC\;1}\;-\;\delta {}^{13}C_{added\;OC\;2}\;}\;\times \;100$$

where δCH4, mixture 1 13 and δCH4, mixture 2 13 are the δ^13^C of CH_4_ originating from the mixtures with the first type of added OC (added OC 1) and the second type of added OC (added OC 2), respectively.

Furthermore, if CH_4_ is originating exclusively from the added OC:

(5) $$f_{added\;OC\;}=\;1,\;and\;\delta {}^{13}{CH}_{4,\;mixture\;1}\;-\;\delta {}^{13}C_{added\;OC\;1}\;=\;\delta {}^{13}{CH}_{4,\;mixture\;2}\;-\;\delta {}^{13}C_{added\;OC\;2}.$$

As recommended by Ye et al. (2016), we used this method only for two added types of OC which have a comparable methanogenic potential in an anoxic sediment. Indeed, if two different types of OC have a comparable methanogenic potential, it implies that the degrading OC is of equivalent quality for methanogens, and in case of a sediment rich in electron acceptors, it indicates that they were consumed at the same speed (Ye et al. 2016). Furthermore, the sediment matrix buffers the abiotic conditions such as pH or redox conditions and in our case, it was taken care that the same microbial inoculum was initially added. All these factors (i.e., microbial community, the environment, and the OC quality) drive the pathways of CH_4_ formation (Sugimoto and Wada 1993; Hornibrook et al. 2000; Conrad et al. 2011). Consequently, when two added types of OC have a comparable CH_4_ production over time, the previous assumption that the overall C fractionation between added OC and CH_4_ will follow the same value over time is likely to be warranted (Ye et al. 2016).

This method is less robust for CO_2_ (Conrad et al. 2012) because CO_2_ results from several reactions (production by fermentation and acetoclastic methanogenesis, consumption by hydrogenotrophic methanogenesis) having different C fractionation (Conrad et al. 2010). Besides, a significant fraction may be dissolved and δ^13^C‐CO_2_ might not be totally representative of δ^13^C‐TCO_2_ because of the C fractionation between gaseous CO_2_ and carbonates (Deuser and Degens 1967). Therefore, only the contribution of added OC to CH_4_ was investigated.

## Results

### TCO_2_, CH_4_, and remaining OC

**Figure 2:**
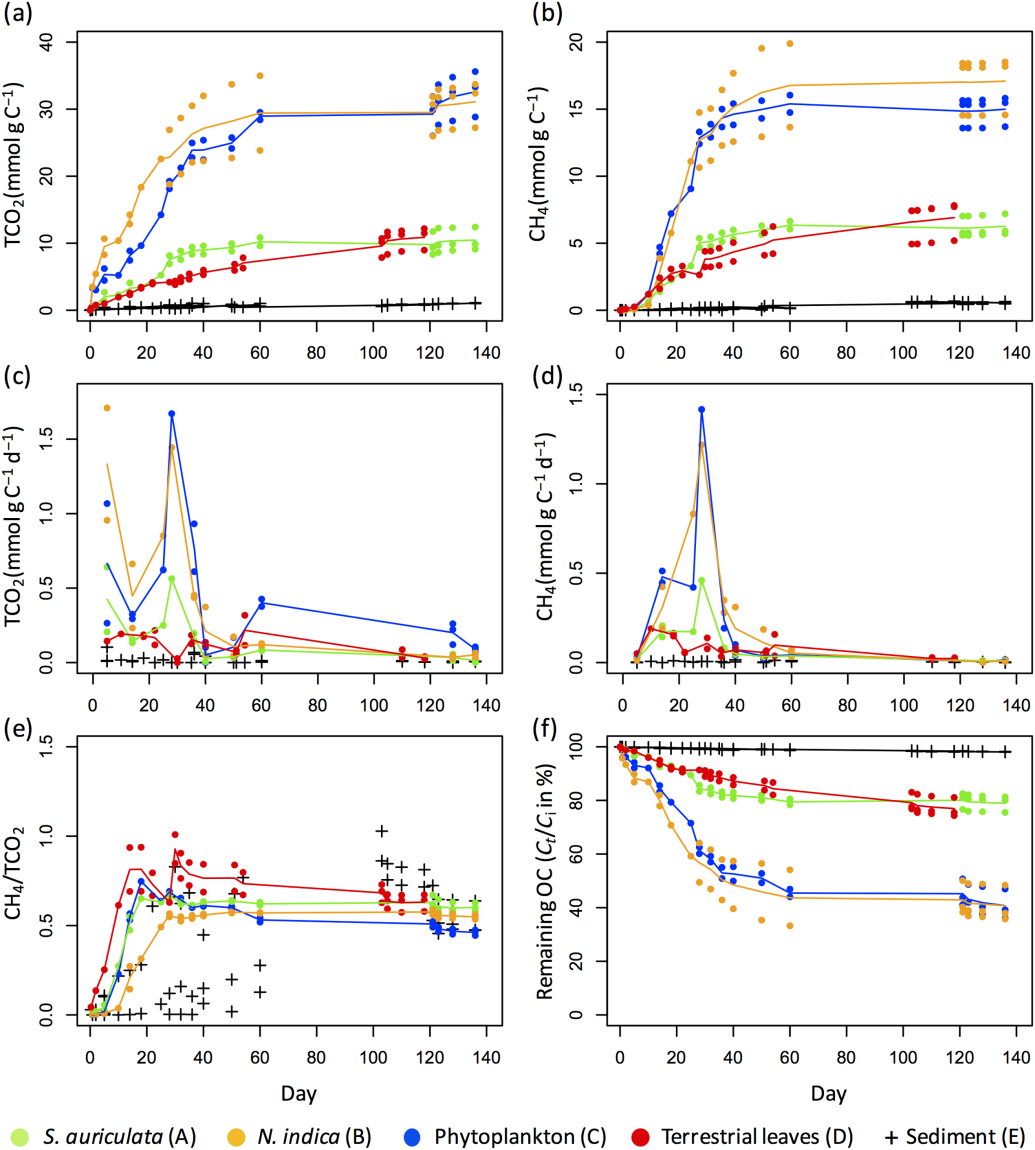
(**a**) TCO_2_ and (**b**) CH_4_ production over time (**c**) TCO_2_ and (**d**) CH_4_ production rates, (**e**) ratio of CH_4_/TCO_2_ production (molar units) and (**f**) fraction of remaining OC for added OC with sediment (treatments A–D) and sediment‐only treatments (treatment E). See Fig. 1 for the description of the different treatments. For added OC with sediment treatments, TCO_2_ and CH_4_ production and production rates, and remaining OC are those attributed to the mineralization of added OC only (see calculations of TCO_2_, CH_4_, and remaining OC in the text).

In the control treatment (only artificial water and sediment inoculum), no CH_4_ production was detected and TCO_2_ production was negligible (total TCO_2_ production < 1 μmol). For all mixture treatments (treatments A–D with added OC + sediment), CH_4_ production started right after the beginning, while there were a few days delay for the sediment‐only treatment (treatment E). The total CH_4_ production (i.e., total cumulative CH_4_ production at the end of the incubation) in the sediment‐only treatment was similar independent of sampling occasion (0.55 ± 0.09 mmol g C^−1^ at day 136 for the sediment sampled in March, and 0.64 ± 0.04 mmol g C^−1^ at day 118 for the sediment sampled in April). CH_4_ and TCO_2_ production of the sediment‐only treatments was very low compared to that of the mixtures treatments (total CH_4_ production between 6.3 mmol g C^−1^ and 17.1 mmol g C^−1^, Fig. 2b). CH_4_ and TCO_2_ production differed among the mixtures, the total CH_4_ and TCO_2_ production for treatments with phytoplankton and *N. indica* being between two and three times higher (15.0 ± 1.1 mmol g C^−1^ and 17.1 ± 2.2 mmol g C^−1^, respectively for CH_4_ and 32.6 ± 3.4 mmol g C^−1^ and 31.1 ± 3.4 mmol g C^−1^, respectively for TCO_2_) that of *S. auriculata* and terrestrial leaves (6.3 ± 0.8 mmol g C^−1^ and 6.9 ± 1.5 mmol g C^−1^, respectively, for CH_4_ and 10.5 ± 1.7 mmol g C^−1^ and 10.9 ± 1.7 mmol g C^−1^, respectively, for TCO_2_, Fig. 2a,b). CH_4_ production followed a similar temporal pattern for the three autoOC types (treatments A–C), it increased quickly and reached a plateau around day 60. For the terrestrial leaves (treatment D), the increase seemed more constant and slower (Fig. 2a,b). CH_4_ and TCO_2_ production rates (Fig. 2c,d), indicated that the decomposition of autoOC was the fastest around day 30 while for alloOC, the production rates were overall slower than for the autoOC types before day 40, and rates decreased slightly and more linearly throughout the incubation. CH_4_ and CO_2_ concentrations measured at day 30 and day 60 in the replicates used for isotope measurements (not shown) were very close to those measured in the replicates used for concentration measurements (Fig. 2), indicating that they followed the same pattern of CH_4_ and CO_2_ production. The ratio of CH_4_/TCO_2_ production was relatively similar for all mixture treatments (treatments A–D) throughout the incubation, it increased during the first 30 d of the incubation to reach 0.5–0.7 for the three autoOC types and 0.9 for the terrestrial leaves, and then stayed relatively constant or slightly decreased to 0.6 for the terrestrial leaves (Fig. 2e).

When comparing the OC decomposition of the different mixture treatments (treatments A–D) using an exponential decay model, the fraction of the degradable pool (parameter *a*, Table 2) was significantly higher for phytoplankton and *N. indica* than for terrestrial leaves and *S. auriculata*. The speed of decay of the degradable pool (parameter *k*, Table 2) was the lowest for terrestrial leaves and relatively close for the three autoOC despite a significant difference between phytoplankton and *N. indica* (Table 2; Fig. 2f). According to the exponential decay model, no further decomposition was predicted after 1 yr of decomposition for the three autoOC, but an additional C loss of 3% was predicted for the terrestrial leaves (Table 2). Even in the treatments with highest extent of OC degradation, about 40% or more of the OC was not degraded over the course of the experiment.

**Table 2:** Parameters and prediction of remaining OC obtained with the exponential decay model of the decomposition of the mixtures with added OC and sediment.

	*a*	*k*	Remaining OC (%)	Predicted remaining OC at 1 yr (%)
*S. auriculata* (A)	0.21 ± 0.01*** b	0.039 ± 0.008*** ab	79 ± 3	79
*N. indica* (B)	0.59 ± 0.01*** a	0.043 ± 0.003*** a	41 ± 7	41
Phytoplankton (C)	0.59 ± 0.01*** a	0.034 ± 0.003*** b	41 ± 6	41
Terrestrial leaves (D)	0.26 ± 0.04*** b	0.016 ± 0.005*** c	77 ± 4	74

*a* and *k* are the parameters (mean ± SE) given by the exponential decay model, *a* is the initial fraction of the degradable pool, 1 − *a* is that of the residual pool (unitless), and *k* is the first‐order decay constant (d^−1^).

Significance levels of the parameters are: *p < 0.05; **p < 0.01; ***p < 0.001; ns, not significant. The different letters after the significance level (a, b, c) indicate that the parameters significantly differ between the mixtures with sediment and added OC.

The fraction of remaining OC (mean ± SD) is given at day 118 for the treatments with terrestrial leaves and at day 136 for the other treatments. Both fractions of remaining OC and predicted remaining OC are in percentage of the initial OC.

### CH_4_ source partitioning

**Figure 3:**
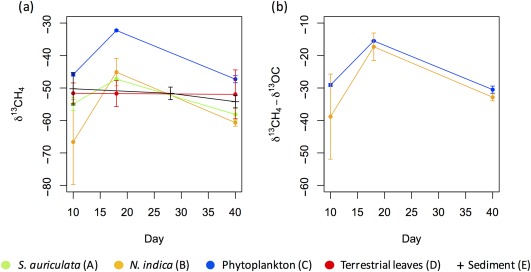
(**a**) δ^13^C of CH_4_ (mean ± 2SD, *n* = 3) produced during the decomposition of sediment with added OC (treatments A–D) and sediment‐only (treatment E). (**b**) δ^13^C of CH_4_ (mean ± 2SD, *n* = 3) produced during the decomposition of *N. indica* or phytoplankton with sediment minus δ^13^C‐OC of *N. indica* and phytoplankton, respectively (i.e., corresponds to δCH4, mixture 13− δCadded OC 13 in Eq. 5).

δ^13^C of CH_4_ was relatively constant and similar for the mixtures with terrestrial leaves and sediment (treatment D) and sediment‐only (E) but varied with time for autoOC + sediment (A–C), with a rapid enrichment in ^13^C at day 18, followed by a decrease in ^13^C at day 40. In contrast to all other types of added OC, autoOC derived from phytoplankton produced higher δ^13^C‐CH_4_ and δ^13^C‐CO_2_ signals (Fig. 3a; Supporting Information Fig. S1). The δ^13^C of CH_4_ derived from phytoplankton + sediment could be compared with that derived from the mixtures of *N. indica* + sediment to estimate the contribution of phytoplankton and *N. indica* to the CH_4_ produced. Indeed, the two types of OC followed the two conditions mentioned in the methods: (1) phytoplankton and *N. indica* had different δ^13^C‐OC (−16.8‰ and −27.8‰, respectively; Table 1), and (2) they had a similar CH_4_ production over time (Fig. 2b,d). The δ^13^C signature of CH_4_ produced in the mixture treatments minus δ^13^C of the added OC (i.e., δCH4, mixture 13− δCadded OC 13 in Eq. (5)) was very close for *N. indica* + sediment and phytoplankton + sediment (Fig. 3b) implying that CH_4_ was mostly originating from the two added OC (Eq. (5)). The δ^13^C‐CH_4_ of *N. indica* + sediment was highly variable between the replicates at day 10 (δ^13^C‐CH_4_ from −78‰ to −55‰), thus $f_{added\;OC\;}$, the contribution of added OC to CH_4_ was only calculated for day 18 and day 40. According to Eq. (4), $f_{added\;OC\;}=$116% ± 33% at day 18 and 121% ± 12% at day 40, meaning that essentially all the CH_4_ produced in these mixtures was derived from the added OC.

## Discussion

### Comparison of CH_4_ production between sediment with added OC and sediment only

This study shows that large CH_4_ production can result from the addition of fresh OC to anoxic sediments, particularly from autoOC, but also from alloOC, within timescales of weeks to months. CH_4_ production from the pre‐existing sediment only (treatment E) was very low compared to the large CH_4_ production resulting from the addition of fresh autoOC (treatments A–C) and alloOC (treatment D; Fig.2b). The sediment seemed to be poor in inorganic electron acceptors, which could outcompete methanogenesis, because there was a very short lag phase before CH_4_ production started in the sediment‐only treatment E (Ye et al. 2016). The low CH_4_ production from sediment only may consequently rather be attributed to a low availability of labile compounds than a high content of inorganic electron acceptors. The large CH_4_ production following the addition of autoOC was expected since several studies demonstrated that autoOC is easily decomposed in anoxic sediments (Schulz and Conrad 1995; Kankaala et al. 2003; Schwarz et al. 2008; West et al. 2012). However, to our knowledge, the high CH_4_ production potential of fresh terrestrial leaves decomposing in lake sediments is a new finding, and West et al. (2012) did not observe a significant difference in CH_4_ production between the sediment without OC addition and the sediment with fresh terrestrial leaves. Our finding is consistent to what Guerin et al. (2008) observed during the anaerobic incubation of terrestrial leaves in soils and relates to the sometimes high CH_4_ emissions measured in freshwater systems with high alloOC inputs (Sollberger et al. 2014). The large CH_4_ production resulting from the addition of all OC types in sediments, even alloOC, is particularly interesting since it suggests that all systems with high OC sedimentation rates and anoxic bottom waters, be it tropical reservoirs with high alloOC sedimentation or eutrophic lakes with high autoOC sedimentation, have the potential to emit substantial amounts of CH_4_.

### Contribution of degradation of added OC to CH_4_ production

The very low CH_4_ production from the sediment‐only incubation in comparison to that of added OC suggested that CH_4_ was mainly fueled by added OC in the mixture treatments. However, this mass balance approach is only valid if the mineralization of sediment OC is not stimulated by the addition of fresh OC (positive priming). The CH_4_ partitioning results derived from isotopic analyses supported the mass balance approach, indicating that CH_4_ production from sediment OC was very low also in presence of added OC ( $f_{added\;OC\;}$ > 100%; Fig. 3b). This shows that a positive priming effect did not occur, or did not visibly increase the sediment contribution to CH_4_ production in comparison to the large contribution of the fresh added OC. Hence, both approaches (mass balance and CH_4_ partitioning) support our first hypothesis that the supply of fresh OC to an anoxic refractory sediment will increase CH_4_ production, and that CH_4_ will be fueled mainly by fresh OC. Our study is the first to partition CH_4_ production in an anoxic sediment, therefore, other studies with different sediment OC reactivity and different availability of inorganic electron acceptors (as electron acceptors can inhibit methanogenesis and be quickly consumed after fresh OC addition, Ye et al. 2016) are needed to further investigate the importance of a priming effect for CH_4_ production in anoxic lake sediments.

The patterns of δ^13^C‐CH_4_ produced during the first 40 d for the mixtures with autoOC sediment (treatments A–C) (Fig. 3a; Supporting Information) were typical to what is observed in anoxic decomposition experiments of soils or sediments with fresh added OC (Sugimoto and Wada 1993; Conrad et al. 2012). The ^13^C‐CH_4_ enrichment at the beginning was followed by a decrease in ^13^C‐CH_4_, due to changes in substrate δ^13^C (i.e., the acetate pool becoming enriched in ^13^C the first weeks, Goevert and Conrad 2009), OC quality and contribution of the different pathways for CH_4_ production (Sugimoto and Wada 1993; Hornibrook et al. 2000). In comparison, δ^13^C‐CH_4_ signature of sediment‐only (treatment E) and terrestrial leaves + sediment (treatment D) varied little (Fig. 3a), possibly because of their low content in labile compounds or because of the progressive and slower decay of the degradable pool.

### Difference in decomposition dynamics between the OC types

To our knowledge, this study is the first comparing the anoxic decomposition and methanogenic potential of the three main types of OC depositing in lake sediments (namely aquatic plant leaves, phytoplankton, and terrestrial leaves). Even though all added OC types were able to fuel methanogenesis, the decomposition dynamics greatly differed between the types of OC that were added to the sediment. We hypothesized that autoOC would decompose faster than alloOC and thus would sustain higher CO_2_ and CH_4_ production rates. The speed of decay of the degradable pool was indeed significantly faster for the mixtures with autoOC (treatments A–C) than for the mixture with terrestrial leaves (treatment D) according to the exponential decay model (parameter k in Table 2). Furthermore, while the autoOC treatments A–C reached a plateau in degradation after 60 d, the degradable pool in terrestrial leaves treatment was not completely depleted at the end of the 118 d incubation (additional C loss of 3% after 1 yr, Table 2). This was further supported by CO_2_ and CH_4_ production rates, indicating that for autoOC the degradable pool was very quickly decomposed (most decomposition occurring around day 30), while for alloOC, CO_2_, and CH_4_ production rates were more constant over time, indicating a more progressive decomposition of the degradable pool (Fig. 2c,d). These different dynamics of decomposition between autoOC and alloOC are in accordance with studies on DOC (Guillemette et al. 2013) or POC (Kristensen and Holmer 2001) decomposition, and may be attributable to lower hydrolysis and/or fermentation rates of the terrestrial OC degradable pool because this fraction is assumed to be chemically more complex and more difficult for enzymes to access due to the lignocellulose structure (Webster and Benfield 1986; Kristensen and Holmer 2001; Dai et al. 2005). Another potential explanation for the slower degradation rate of alloOC compared to autoOC may be that the alloOC treatment was composed of 17 species, each potentially having different degradability, and hence leading to an apparently more progressive decomposition. Overall, the observed differences in degradation dynamics between autoOC and alloOC may have an important implication. A high pulse of CH_4_ production fueled by the rapid anoxic decomposition of autoOC is more likely to lead to oversaturation of CH_4_ in sediment pore water and therefore CH_4_ ebullition, which is the most important CH_4_ emission pathway to the atmosphere. For the same quantity of OC, the comparatively slower and more constant production of CH_4_ fueled by the anoxic decomposition of terrestrial leaves is more likely to stimulate CH_4_ diffusion from the sediment, a significant share of which will be microbially oxidized to CO_2_.

### Difference in decomposition yield between the OC types

Even if the exponential decay model and the production rates indicated a quicker decomposition for autoOC than alloOC, we did not find higher decomposition yield (i.e., overall extent of OC decomposition) and total CH_4_ production for autoOC than for alloOC (parameter a in Table 2, Fig. 2b,f). Indeed, the phytoplankton had higher decomposition yield and total CH_4_ production than the terrestrial leaves (41% and 77% of OC remaining for phytoplankton and terrestrial leaves, respectively), as hypothesized, but one aquatic plant had similar decomposition yield as the terrestrial leaves (79% of OC remaining for *S. auriculata*), and the other aquatic plant similar decomposition yield as the phytoplankton (41% for *N. indica*, Fig. 2f, Table 2). The higher decomposition yield and total CH_4_ production from phytoplankton OC compared to terrestrial leaves are consistent with the results of West et al. (2012) comparing the decomposition of these two types of OC in anoxic lake sediments. Similarly, several studies demonstrated a higher preservation of terrestrial OM in lake sediments (Sobek et al. 2009; Guillemette et al. 2016) and a positive relationship between lakes chlorophyll *a* concentration and CH_4_ emissions (Deemer et al. 2016; DelSontro et al. 2016). While other studies have reported variable extents of CH_4_ production from the decomposition of aquatic plants in lakes (Kankaala et al. 2003) or coastal wetland sediments (Vizza et al. 2017), our study is the first to report that the degradation of aquatic vascular plants to CH_4_ spans all the way from the comparatively low CH_4_ production of terrestrial leaves to the high CH_4_ production of phytoplankton (Fig. 2b). These different extent of degradation to CH_4_ between the two species may be attributable to different contents in refractory compounds, such as a high content in waxes for *S. auriculata* (Barthlott et al. 2009; Mortillaro et al. 2016) or a low content in structural compounds for *N. indica* (Esteves and Barbieri 1983).

The highly different degradation behavior of the two aquatic vascular plant species in this study (treatments A and B; Fig. 2, Table 2) suggests that the extent to which C fixed by aquatic plants is emitted as GHG or buried as OC in sediment could more generally differ between aquatic vegetation types. This could have consequences for lake and reservoir management, and needs to be further explored. For a more comprehensive view of the effect of different aquatic vegetation types on greenhouse gas emissions, other processes than OC decomposition would need to be taken into account such as primary productivity, the quantity of substrates provided to methanogens (Whiting and Chanton 1993), or CH_4_ rhizospheric oxidation (Ribaudo et al. 2012; Attermeyer et al. 2016). Furthermore, other factors than the type of OC might act on CH_4_ production in freshwater sediments, such as the sediment content in electron acceptors, and temperature, and should be investigated to better understand and predict CH_4_ production in freshwaters.

### Implications

The addition of fresh OC to anoxic sediment resulted in large CH_4_ production, both for autoOC and alloOC. The three types of autoOC could sustain higher CH_4_ production rates than alloOC, corresponding to a higher potential to induce CH_4_ supersaturation in sediment pore water and stimulate CH_4_ ebullition. Our results consequently indicate that all systems with high sedimentation rates can be CH_4_ emitters, especially if they have anoxic bottom waters and high internal primary productivity. Such systems (e.g., eutrophic lakes and reservoirs), are generally regarded as C sinks, because of frequent CO_2_ undersaturation and/or high OC burial (Pacheco et al. 2013; Anderson et al. 2014), but if a significant part of the sedimenting OC is returned to the atmosphere as CH_4_, the CH_4_ emissions could offset the C sink in terms of global warming potential (Bastviken et al. 2011; Supporting Information). As most of CH_4_ is released through ebullition and plant transport (Schütz et al. 1991; Wilkinson et al. 2015), a challenge for future studies would to better quantify the fraction of CH_4_ that is returned to the atmosphere through these two pathways.

## Conflict of Interest

None declared.

## Supporting information

Supporting Information
