# Supplementary material for: Large but variable methane production in anoxic freshwater sediment upon addition of allochthonous and autochthonous organic matter
Source: Limnol Oceanogr. 2018 Feb 6;63(4):1488–501. doi: 10.1002/lno.10786 (PMC6108407; doi:10.1002/lno.10786)
Supplement: Supplementary file 1 — Supporting Information [file LNO-63-1488-s001.docx]

**Supplementary information**

**Large but variable methane production in anoxic freshwater sediment upon addition of allochthonous and autochthonous organic matter**

Charlotte Grasset^1*^, Raquel Mendonça^1,2^, Gabriella Villamor Saucedo^1,2^, David Bastviken^3^, Fabio Roland ^1^, Sebastian Sobek^2^

~~
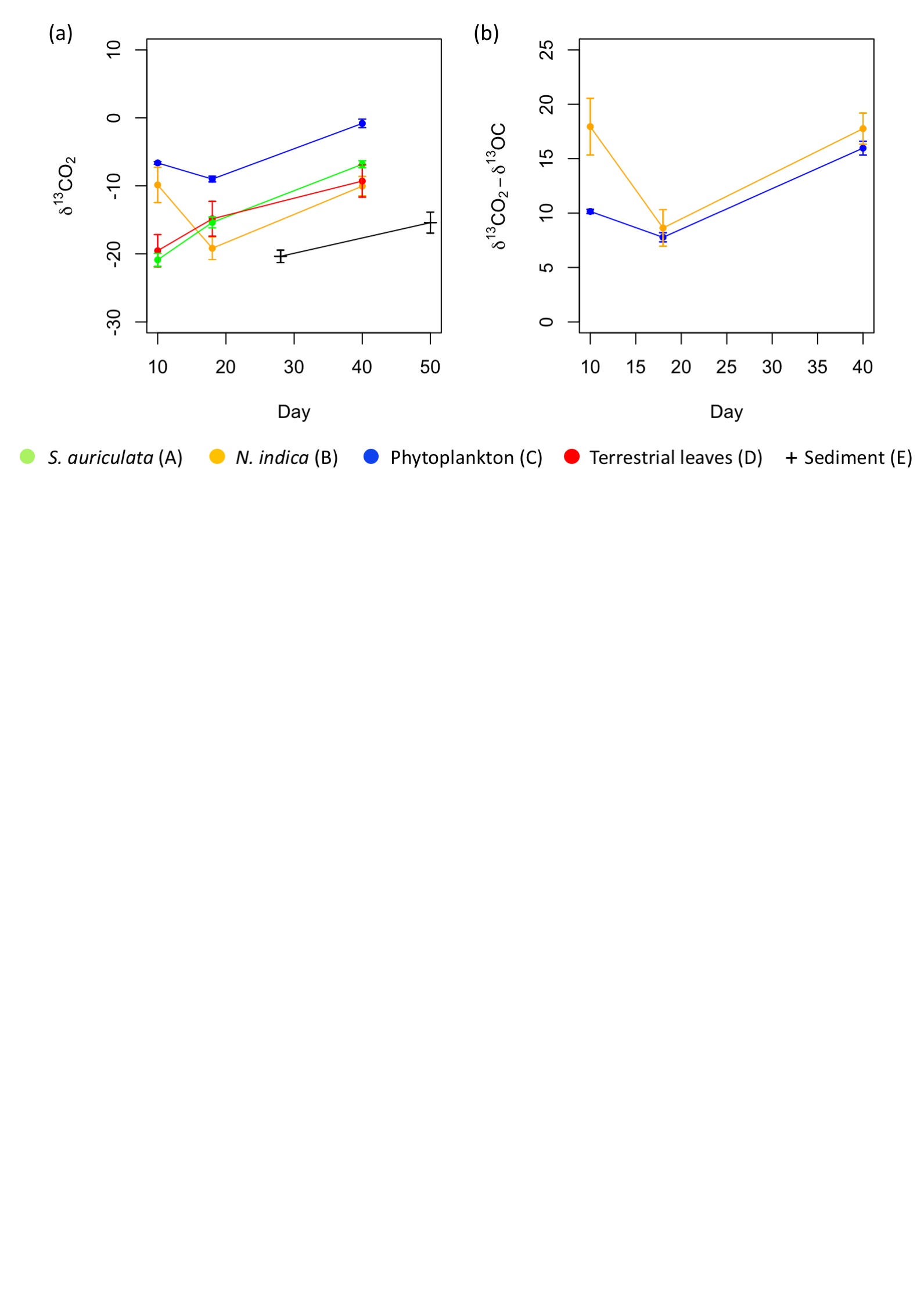
~~

**Fig. S1** (a) δ^13^C of CO_2_ (mean ± 2SD, n=3) produced during the decomposition of sediment with added OC (treatments A-D) and sediment-only (treatment E). (b) δ^13^C of CO_2_ (mean ± 2SD, n=3) produced during the decomposition of *N. indica* or phytoplankton with sediment minus δ^13^C-OC of *N. indica* and phytoplankton, respectively.

**δ^13^C-OC signatures added OC and sediment**

The δ^13^C-OC signatures of the added OC types (Table 1) were typical to what is usually observed for C3 terrestrial plants and for the same aquatic plants (between -32 and -22 ‰ for C3 terrestrial plants, Finlay and Kendall 2008; between -24 and -27 ‰ for *N. indica* and between -28 and -29 ‰ for *Salvinia sp*., Mendonça et al. 2013). The high value of δ^13^C of phytoplankton, sampled during a phytoplanktonic bloom, was attributed to the enrichment in ^13^C of DIC due to a high primary production (Gu et al. 2006; de Kluijver et al. 2014). The surface sediment had a higher δ^13^C-OC signature than the C3 terrestrial plant leaves sampled close to the reservoir, because plants in C4 (mainly grasses with a δ^13^C-OC around -13 ‰, Finlay and Kendall 2008) were also present around the reservoir and contributed to sediment organic carbon (Isidorova, A et al., in preparation).

**Estimation of the warming potential effect of the different types of OC**

The variability in decomposition yield and total CH_4_ production of the different types of OC could lead to contrasting global warming potential effects, depending on the quantity of CH_4_ that will be transported to the atmosphere (Table S1). The estimation of the warming potential was calculated as the C lost as CO_2_ or CH_4_ minus the predicted remaining OC after 1 year, with all fluxes expressed as CO_2_-equivalents. We assumed that at a low CH_4_ transport efficiency, only 10% of the CH_4_ produced is being returned to the atmosphere and the other 90% is being oxidized into CO_2_ (Segers 1998) while at a high CH_4_ transport efficiency, 60% of CH_4_ is returned to the atmosphere (Wilkinson et al. 2015). At a low CH_4_ transport efficiency, only the anoxic decomposition of phytoplankton and *N. indica* would result in a positive effect on radiative forcing in the atmosphere. However, at a high CH_4_ transport efficiency, which is typical for highly ebullitive sediments or in zones where CH_4_ transport by plant is predominant (Schütz et al. 1991; Chanton 2005), all treatments would have a positive effect on radiative forcing in the atmosphere (Table S1). These back-on-the-envelope calculations of course are highly uncertain (e.g. by assuming that the degradation of OC will cease after 1 yr, that all degradation occurs anaerobically, or that DOC release from anoxic sediment is negligible, Peter et al. 2016). However, they illustrate that C fixed by both aquatic and terrestrial plants, as well as by phytoplankton, can be returned to the atmosphere partially as CH_4_ and therefore cause an amplification in radiative forcing, particularly if CH_4_ is transported by bubbles or through plants.

**Table S1.** Estimation of the warming potential related to the decomposition of the different added OC.

|  | Estimation of warming potential at 1 year (%) |
| --- | --- |
| *S. auriculata* (A) | -36 - 71 |
| *N. indica* (B) | 76 - 360 |
| Phytoplankton (C) | 68 - 319 |
| Terrestrial leaves (D) | -20 - 119 |

CH_4_ and CO_2_ loss after one year are calculated from the predicted remaining OC after one year and the CH_4_: CO_2_ ratio at the end of the experiment. CH_4_ is expressed in CO_2_ equivalents to take into account its warming potential (at 100 years, CH_4_=28 CO_2_ equivalents, IPCC 2014). The estimation is given in % of the initial OC.

**References**

Chanton, J. P. 2005. The effect of gas transport on the isotope signature of methane in wetlands. Org. Geochem. **36:** 753-768.

de Kluijver, A., P. L. Schoon, J. A. Downing, S. Schouten, and J. J. Middelburg. 2014. Stable carbon isotope biogeochemistry of lakes along a trophic gradient. Biogeosciences **11:** 6265-6276.

Gu, B., A. D. Chapman, and C. L. Schelske. 2006. Factors controlling seasonal variations in stable isotope composition of particulate organic matter in a softwater eutrophic lake. Limnology and Oceanography **51:** 2837-2848.

Finlay, J. C., and C. Kendall. 2008. Stable Isotope Tracing of Temporal and Spatial Variability in Organic Matter Sources to Freshwater Ecosystems, p. 283-333. Stable Isotopes in Ecology and Environmental Science. Blackwell Publishing Ltd.

IPCC. 2014. Climate Change 2014: Synthesis Report. Contribution of Working Groups I, II and III to the Fifth Assessment Report of the Intergovernmental Panel on Climate Change.

Mendonça, R. and others 2013. Bimodality in stable isotope composition facilitates the tracing of carbon transfer from macrophytes to higher trophic levels. Hydrobiologia **710:** 205-218.

Peter, S., A. Isidorova, and S. Sobek. 2016. Enhanced carbon loss from anoxic lake sediment through diffusion of dissolved organic carbon. Journal of Geophysical Research: Biogeosciences **121:** 1959-1977.

Schütz, H., P. Schröder, and H. Rennenberg. 1991. 2 - Role of Plants in Regulating the Methane Flux to the Atmosphere, p. 29-63. *In* T. D. S. A. H. A. Mooney [ed.], Trace Gas Emissions by Plants. Academic Press.

Segers, R. 1998. Methane production and methane consumption: a review of processes underlying wetland methane fluxes. Biogeochemistry **41:** 23-51.

Wilkinson, J., A. Maeck, Z. Alshboul, and A. Lorke. 2015. Continuous Seasonal River Ebullition Measurements Linked to Sediment Methane Formation. Environmental Science & Technology **49:** 13121-13129.
